# A Sensitive and Quantitative Polymerase Chain Reaction-Based Cell Free *In Vitro* Non-Homologous End Joining Assay for Hematopoietic Stem Cells

**DOI:** 10.1371/journal.pone.0033499

**Published:** 2012-03-20

**Authors:** Lijian Shao, Wei Feng, Kyung-Jong Lee, Benjamin P. C. Chen, Daohong Zhou

**Affiliations:** 1 Division of Radiation Health, Department of Pharmaceutical Sciences, University of Arkansas for Medical Sciences, Little Rock, Arkansas, United States of America; 2 Division of Molecular Radiation Biology, Department of Radiation Oncology, University of Texas Southwestern Medical Center, Dallas, Texas, United States of America; St. Georges University of London, United Kingdom

## Abstract

Hematopoietic stem cells (HSCs) are responsible for sustaining hematopoietic homeostasis and regeneration after injury for the entire lifespan of an organism. Maintenance of genomic stability is crucial for the preservation of HSCs, which depends on their efficient repair of DNA damage, particularly DNA double strand breaks (DSBs). Because of the paucity of HSCs and lack of sensitive assays, directly measuring the ability of HSCs to repair DSBs has been difficult. Therefore, we developed a sensitive and quantitative cell free *in vitro* non-homologous end joining (NHEJ) assay using linearized plasmids as the substrates and quantitative polymerase chain reaction (qPCR) technique. This assay can sensitively detect DSB repair via NHEJ in less than 1 µg 293T cell nuclear proteins or nuclear extracts from about 5,000 to 10,000 human BM CD34^+^ hematopoietic cells. Using this assay, we confirmed that human bone marrow HSCs (CD34^+^CD38^−^ cells) are less proficient in the repair of DSBs by NHEJ than HPCs (CD34^+^CD38^+^ cells). In contrast, mouse quiescent HSCs (Pyronin-Y^low^ LKS^+^ cells) and cycling HSCs (Pyronin-Y^hi^ LKS^+^ cells) repaired the damage more efficiently than HPCs (LKS^−^ cells). The difference in the abilities of human and mouse HSCs and HPCs to repair DSBs through NHEJ is likely attributed to their differential expression of key NHEJ DNA damage repair genes such as *LIG4*. These findings suggest that the qPCR-based cell free *in vitro* NHEJ assay can be used to sensitively measure the ability of human and mouse HSCs to repair DSBs.

## Introduction

Hematopoietic stem cells (HSCs) are responsible for sustaining hematopoietic homeostasis and regeneration after injury for the entire lifespan of an organism [1]. Maintenance of genomic stability has been shown to be crucial for the preservation of HSCs, because mice that are deficient in the expression of various DNA damage repair genes exhibit premature exhaustion of HSCs [2], [3]. In addition, HSCs are long living cells that represent ideal cellular targets for acquisition of multiple mutations, leading to cell transformation and leukemia/lymphoma. Therefore, deficiency in the repair of DNA damage by HSCs not only leads to premature HSC exhaustion and bone marrow (BM) failure but also causes cancer and leukemia predisposition as shown in several animal models [1]. For example, mutational inactivation of the “*ataxia telangiectasia mutated* (*ATM*)” gene in mice causes progressive BM failure and a high incidence of lymphoma [4], [5]. Similarly, it was reported that mice with a *Rad50* hypomorphic mutation (*Rad50^S/S^* mice) exhibited progressive HSC failure and cancer predisposition [6]. In addition, HSCs from *LIG4 (LIG4^Y288C^)* hypomorphic mutated and *KU80* ^−/−^ mice exhibited a dramatic decrease in their ability to self-renew and reconstitute hematopoiesis after transplantation [7], [8].

DNA double-strand breaks (DSBs) are considered as the most detrimental genotoxic DNA lesions, which can arise endogenously from an increase in ROS production and collapse of replication forks or exogenously from exposure to various DNA damaging agents, including ionizing radiation (IR) [9]. Failure to rejoin DSBs can lead to gene deletion and even cell death, whereas the misrepair of DSBs may give rise to chromosome translocation and cell transformation. As such, cells have evolved two major repair pathways to repair DSBs: non-homologous end joining (NHEJ) [10] and homologous recombination (HR) [11]. NHEJ repairs DSBs by re-ligating two DNA broken ends, which is often imprecise and thus error prone because the DNA ends frequently require modification before they can be rejoined, leading to deletions or insertions at the break site [10]. In contrast, HR uses sister chromatids as templates to faithfully repair the damage [11]. Since sister chromatids are only available when cells are in the late S and G_2_ phases, only proliferating cells are able to repair DSBs through this high fidelity repair pathway.

However, whether HSCs can efficiently repair DSBs remain controversial. For example, Milyavsk et al. recently showed that HSCs from human cord blood repaired ionizing radiation (IR)-induced DSBs much slower than hematopoietic progenitor cells (HPCs) [12]. In contrast, at the same time Mohrin et al. reported that quiescent HSCs from adult mouse BM repaired IR-induced DSBs more efficiently than proliferating HPCs primarily via the NHEJ pathway [13]. Several possibilities have been raised to explan the difference in the abilities of human and mouse HSCs to repair DSBs [14], [15]. However, the lacking of an assay that can be used to directly and sensitively measure NHEJ activities in the rare population of stem cells such as HSCs has been a big challenge for studying the difference of human and mouse HSC NHEJ activities. Therefore, we developed a qPCR-based cell free *in vitro* NHEJ assay to meet the need. We found that this assay could sensitively detect DSB repair via NHEJ in less than 1 µg 293T cell nuclear proteins or nuclear extracts from about 5,000 to 10,000 human BM CD34^+^ hematopoietic cells. Using this assay, we confirmed that human HSCs (CD34^+^CD38^−^ cells) are less proficient in the repair of DSBs by NHEJ than HPCs (CD34^+^CD38^+^ cells). In contrast, mouse quiescent HSCs (Pyronin-Y^low^ lin^−^c-kit^+^sca1^+^ cells or PY^low^LKS^+^ cells) and cycling HSCs (Pyronin-Y^hi^ lin^−^c-kit^+^sca1^+^ cells or PY^hi^LKS^+^ cells) repaired the damage more efficiently than cycling HPCs (lin^−^c-kit^+^sca1^−^ cells or LKS^−^ cells). The difference in the abilities of human and mouse HSCs and HPCs to repair DSBs through NHEJ is likely attributed to their differential expression of key DNA damage repair genes such as *LIG4*. These findings suggest that the qPCR-based cell free *in vitro* NHEJ assay can be used to sensitively measure the ability of HSCs to repair DSBs. This assay can also be applied to study DSB repair in other populations of rare tissue stem cells. In addition, it could help us to gain more insights into the mechanisms by which HSCs and tissue stem cells maintain their genomic stability.

## Materials and Methods

### Reagents and cells

pDsRed2ER plasmid was ordered from Clontech (Mountain View, CA). BglII and SmaI restriction enzymes were purchased from New England Biolabs (Ipswich, MA). Alexa Fluor® 647-conjugated anti-human CD34 antibody, PE-conjugated anti-human CD38 antibody, Alexa Fluor® 488-conjugated anti-mouse Sca-1, APC-conjugated anti-c-Kit were purchased from BioLegend (San Diego, CA). 293T cells were ordered from ATCC (Manassas, VA). Human BM CD34^+^ hematopoietic cells were purchased from Lonza (Walkersville, MD). Immortalized wild type (WT), *DNA-PKcs* ^−/−^, *Ku70* ^−/−^, and *Ku80* ^−/−^ mouse fibroblast cell lines were cultured and purified DNA-PKcs from human HeLa cells were prepared as previously described [16], [17].

### Animals

Male C57BL/6-Ly-5.2 mice and *PRKDC* (*DNA-PKcs*) null SCID mice were purchased from Jackson Lab (Bar Harbor, MA) and housed at University of Arkansas for Medical Sciences (UAMS) AAALAC certified animal facilities. They received food and water *ad libitum*. All mice were used at approximately 8–12 weeks of age. The Institutional Animal Care and Use Committees of UAMS approved all experimental procedures used in this study (approval number: AUP3057 & 3093).

### Preparation of nuclear protein extracts

Cells were seeded into 100 mm tissue culture dishes at a density of 5×10^6^ per dish. They allowed to growth overnight in Dulbecco's modified Eagle's minimum (DMEM) supplemented with 10% fetal bovine serum (Alanta Biologicals, Lawrenceville, GA), 100 U/ml penicillin and 100 µg/ml streptomycin in a humidified incubator (95% air/5% CO_2_) at 37°C to reach about 80% confluence. After washed once with 10 ml PBS, they were harvested in 1 ml of PBS containing 2% FBS using a rubber scraper. The cells were transferred to a 1.5 ml microcentrifuge tube and centrifuged at 2000× *g* for 5 min. The cell pellet was resuspended in 300 µl buffer I containing 10 mM HEPES, 10 mM KCl, 1.5 mM MgCl2, 500 µM PMSF, 1 mM DTT and protease inhibitor mixture (Cat# p-8340, Sigma, St. Louis, MO) and incubated on ice for 15 min. Six µl of 10% Nonidet P-40 were added to the cell lysates and mixed by vortex for 5 sec. Nuclei were isolated by centrifugation of the lysates at 6,000× *g* for 5 min. The supernatant (containing cytoplasmic proteins) was transferred to another chilled tube and the nuclear pellet was resuspended in 50 µl of buffer II containing 20 mM HEPES, 420 mM NaCl, 1.5 mM MgCl_2_, 0.2 mM EDTA and 25% v/v Glycerol. After 40-min incubation, the nuclear extracts were collected by centrifugation at 13,000× *g* for 10 min at 4°C. Nuclear proteins were also extracted from different numbers of human and mouse HSCs and HPCs in a similar manner as described above but with less lysis and extraction buffers. The protein concentration of the extracts was determined by a Bradford assay kit (Bio-Rad, Hercules, CA) according to the manufacturer's instruction. The nuclear extracts were immediately used or stored at −80°C.

### Standard cell free *in vitro* NHEJ activity assay

**Figure 1:**
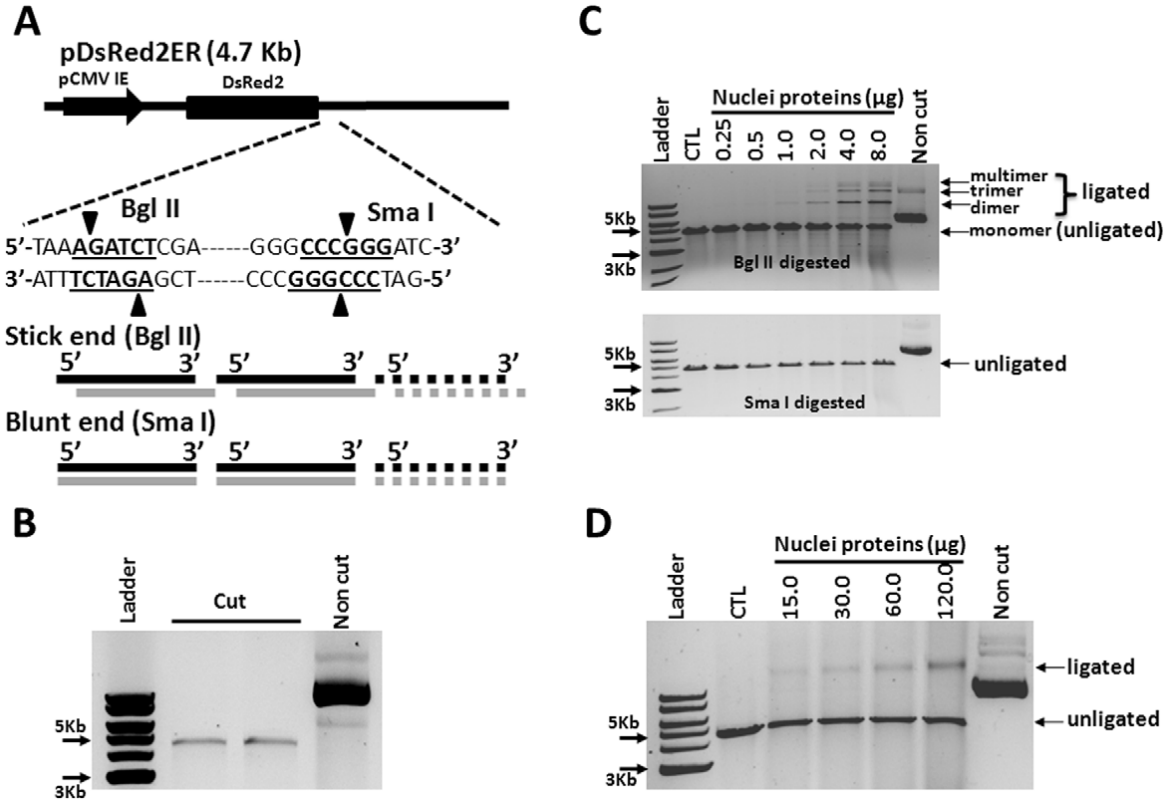
Linearized pDsRed2ER plasmids with cohesive ends are better substrates for the cell free *in vitro* NHEJ assay than those with blunt ends. **A.** A diagram to illustrate the strategy for how to prepare linearized pDsRed2ER plasmid substrates with cohesive and blunt ends by enzymatic digestion with Bgl II and Sma I, respectively. **B.** Confirmation of the purity of the linearized pDsRed2ER plasmid substrates by agarose gel electrophoresis. A representative agarose gel electrophoresis image is shown. **C.** Representative agarose gel electrophoresis images to illustrate the formation of various NHEJ products after the Bgl II (the upper panel)- and Sma I (the lower panel)-digested linearized pDsRed2ER plasmid substrates were incubated with increasing concentrations (0.25 µg to 8.0 µg) of 293T cell nuclear protein extracts. **D.** A representative agarose gel electrophoresis image to illustrate the formation of ligated NHEJ products after the Sma I-digested linearized pDsRed2ER plasmid substrates were incubated with higher concentrations (15 µg to 120.0 µg) of 293T cell nuclear protein extracts.

Linearized pDsRed2ER plasmid substrates with either cohesive or blunt ends were generated by the digestion with BglII or SmaI and purified using Qiagen gel extraction kit (Valencia, CA) after separation on 0.7% agarose gels by electrophoresis to remove residual uncut circular plasmids (Fig. 1A & B). An optimized concentration (150 ng) of the purified linearized pDsRed2ER plasmid substrates was incubated with various concentrations of nuclear protein extracts in the end joining reaction buffer (1 mM ATP, 0.25 mM dNTP, 25 mM Tris acetate, 100 mM potassium acetate, 10 mM magnesium acetate, 1 mM DTT at pH 7.5) in a 30-µL reaction volume for 1 h at 37°C. The reaction mixture was deproteinized with 1 mg/ml proteinase K at 65°C for 30 min and then was separated by electrophoresis on a 0.7% agarose gel for 3 h at 90 V. DNA was detected using a gel imaging system (Alpha Innotech Corp. Santa Clara, CA) after ethidium bromide staining.

### qPCR-based cell free *in vitro* NHEJ activity assay

**Figure 2:**
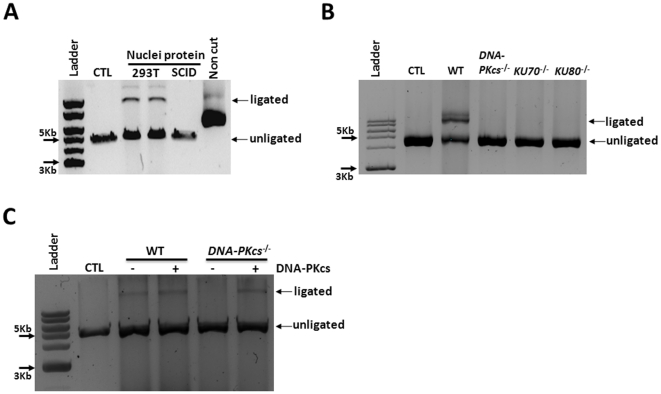
The formation of the NHEJ products depends on DNA-PKcs, Ku70 and Ku80. **A–B.** Nuclear protein extracts from 293T cells and DNA-PKcs null SCID mouse BM cells (SCID) (**A**) and those from immortalized wild type (WT), *DNA-PKcs* ^−/−^, *Ku70* ^−/−^, and *Ku80* ^−/−^ mouse fibroblast cell lines (**B**) were incubated with the Bgl II-digested linearized pDsRed2ER plasmid substrates. **C.** Nuclear protein extracts from immortalized wild type (WT) or *DNA-PKcs* ^−/−^ were complemented with 0.24 ng DNA-PKcs purified from HeLa cells and then incubated with the Bgl II-digested linearized pDsRed2ER plasmid substrates. The formation of various NHEJ products was analyzed by agarose gel electrophoresis. A representative agarose gel electrophoresis image is shown. CTL means no nuclear proteins added to the assay.

Linearized pDsRed2ER plasmid substrates (150 ng) were incubated with nuclear protein extracts as described above. The resulting ligated DNAs were diluted with 10 mM pH 8.5 Tris buffer (1∶10,000). One µL of the diluted DNAs was amplified using three pairs of primers, e.g. Ds-F1 and Ds-R2, Ds-F1 and Ds-R3, and Ds-F2 and Ds-R2 designed for the head-to-tail (H-T), head-to-head (H-H), and tail-to-tail (T-T) ligated DNAs, respectively, by qPCR as described below (Fig. 2B). Amplifications from Ds-F1 and Ds-R1 primers were used as an internal control. All primers' sequences were listed in Table 1. For each sample, SYBR Green real-time qPCR was conducted in duplicate or triplicate in 96-well plates using an ABI StepOne plus real time PCR System (Applied Biosystems, Foster City, CA). The 20 µL PCR reaction was prepared as follow: 10 µL of 2× SYBR Green PCR master mix, 0.4 µL of 10 µM of appropriate forward and reverse primers, 7.6 µL RNase-free water, and 2 µL DNA template. A negative control (digested plasmid DNA without nuclear protein) and a positive control (undigested plasmid DNA) were also included for each qPCR reaction. The qPCR was performed with 10 min at 95°C, followed by 40 cycles of 15 s at 95°C and 1 min at 60°C. A dissociation melting curve was generated using thermal conditions from 60°C to 95°C. An arbitrary unit was calculated by the comparative C_T_ method according to the C_T_ values of the internal control as described previously [18].

**Table 1:** Sequences for the primer used in the in vitro qPCR NHEJ assay.

Bgl II generated linear plasmids	Forward sequences	Reverse sequences
Ds-F1	5-TCTCGAGCTCAAGCTTCGAATTCTGCAGTC-3	
Ds-F2	5-GGCGGCCACTACCTGGTGGAGTTCAAGTC-3	
Ds-F3	5-TGTACGATCTCAGGAACAGGTGGTGGCG-3	
Ds-R1		5-TACAGCTCGTCCTTCTTGTACTAGCTCAG-3
Ds-R2		5-TGGCTGATTATGATCAGTTATCTAGATC-3
Ds-R3		5-GAGGGCCGCCACCACCTGTTCCTGAGATC-3

### Western blot analysis

Total cell lysates and cytoplasmic and nuclear protein extracts were separated on a 12% SDS–PAGE and transferred onto a nitrocellulose membrane. Immunoblotting was conducted using 1∶1000 anti-Lamin A/C primary antibody (Cat#2032, cell signaling, Danvers, MA) and 1∶10,000 goat anti-rabbit secondary antibody (Jackson ImmunoResearch Labs., West Grove, PA). Immunocomplexes were detected using Amersham ECL Western Blotting Detection Reagents (GE Healthcare, Piscataway, NJ) and exposed to ECL Plus film. After detection, the membrane was stripped and reprobed with anti-β-actin antibody (Cat# SC-1616, Santa Cruz Biotechnology, Santa Cruz, CA) to control for cytoplasmic protein contamination.

### Flow cytometry and cell sorting

Human BM CD34^+^ hematopoietic cells were stained with Alexa Fluor® 647-conjugated anti-CD34 and PE-conjugated anti-CD38 antibodies. Dead cells were excluded by staining with Propidium Iodide (PI). Human CD34^+^CD38^−^ HSCs and CD34^+^CD38^+^ HPCs were sorted using an Aria II cell sorter. To isolate mouse HPCs (LKS^−^ cells), cycling HSCs (PY^hi^LKS^+^ cells) and quiescent HSCs (PY^low^LKS^+^ cells), mouse BM Lin^−^ cells were prepared as previously described [18]. They were blocked with anti-CD16/32 antibody and then stained with Alexa Fluor® 488-conjugated anti–Sca1 antibody and APC-conjugated anti–c-Kit antibody followed by incubation with PY (1 µg/ml) for 15 min at 37°C in HBSS medium containing 10% FCS, 10 mM HEPES, 1 mg/ml glucose, and 50 µg/ml Verapamil. HPCs, PY^hi^LKS^+^ and PY^low^LKS^+^ cells were sorted immediately using an Aria II cell sorter [19].

### Quantitative reverse transcriptase PCR (qRT-PCR)

Total RNA was isolated using the Qiagen RNeasy Mini Kit (Valencia, CA) according to the manufacturers' instructions. RNA yield and quality were determined by measuring absorbencies at 260 nm and 280 nm, respectively. First-strand cDNA was synthesized in a final volume of 20 µl using the Superscript III First-Strand Synthesis System (Invitrogen, Carlsbad, CA). Quantitative PCR (qPCR) analyses were performed using a SYBR Green mix on an ABI StepOne Plus Real-Time PCR System (Applied Biosystems, Foster City, CA). Human glyceraldehyde-3-phosphate dehydrogenase (GAPDH) and mouse hypoxanthinephophoribosyltransferase (HPRT) transcripts were used as housekeeping internal references for human and mouse mRNA, respectively. The expression of various DSB repair genes were calculated by the comparative C_T_ method as described previously [18]. The sequences for all the primers used in the qRT-PCR assays are listed in Table 2.

**Table 2:** Primer sequences used for real time RT-PCR.

Genes	Forward sequences	Reverse sequences
human *ATM*	5-GATCTTGTGCCTTGGCTACAGATTG-3	5-ATGTCGCTGTTGGGGTAGAAGCTGAG-3
human *DNA-PKcs*	5-GGTGAACTTAAGACCCAGATGACATC-3	5-TCCCTTGAAGTCTGGGGATCTTCTTC-3
human *KU70*	5-GTGATCTCCGAGATACAGGCATCTTC-3	5-TCCTCAAAGTGAACCCTGAGGTCCTC-3
human *KU80*	5-TTCTCAACAGGCTGACTTCCTGGATG-3	5-TGAGGTCAGTGAATATTTCAATATGC-3
human *Ligase4*	5-TCTTCAACTTATAACTCAGAGTTCAG-3	5-TGTAGTGACATTATGCAACTCAGCAG-3
human *XRCC4*	5- CCTGAAAGATGTCTCATTCAGACTTG-3	5- TGAACATCATTCCAATCTCTCAGAAG-3
human *GAPDH*	5-CGGAGTCAACGGATTTGGTCGTAT-3	5-AGCCTTCTCCATGGTGGTGAAGAC-3
mouse *ATM*	5-TGTGTATACATACCAAGCATACGC-3	5-CTGGATGTACTCCGTGATCCAATG-3
mouse *DNA-PKcs*	5-GAATTGCATTAGTGCTGTGGTGCAC-3	5-GTACGTAGGTACTTTCCACCTGC-3
mouse *KU70*	5- AAGATTTGGACAACCCAGGCGCTAAG -3	5-AGCTGGACGTCGCTGAAGAGGTTGGC-3
mouse *KU80*	5-GAGGACACTATTCAAGGGTACCG-3	5-GCAACAGCTGCCGCCTCATCATC-3
mouse *Ligase4*	5-GAGTTGCACAACGTCACCACAGATC-3	5-CATGTCCTTCTCCACACGCTCCAC-3
mouse *XRCC4*	5-AGTCCAGCAGCTGGAGGAGAGTAC-3	5-TCATCCTTATGAAGAGTTTCTGCAGC-3
mouse *HPRT*	5-AGCAGTACAGCCCCAAAATGGTTA-3	5-TCAAGGGCATATCCAACAACAAAC-3

### Statistical analysis

The data were analyzed by analysis of variance (ANOVA). In the event that ANOVA justified post hoc comparisons between group means, these were conducted using the Student-Newman-Keuls test for multiple comparisons. For experiments in which only single experimental and control groups were used, group differences were examined by unpaired Student *t* test. Differences were considered significant at *P<0.*05. All of these analyses were done using GraphPad Prism from GraphPad Software (San Diego, CA).

## Results

### Linearized plasmids with cohesive ends are better substrates for the cell free *in vitro* NHEJ assay than those with blunt ends

Linearized pDsRed2ER plasmid substrates with cohesive and blunt ends were generated by the digestion with BglII and SmaI, respectively, and then purified as shown in Fig. 1A & B. After purification, they were incubated with different concentrations of 293T cell nuclear protein extracts. The formation of circular and linear dimeric, trimeric, and multimeric NHEJ DNA products was analyzed by gel electrophoresis. It was found that the circular NHEJ DNA products were barely detectable with both substrates under the experimental conditions. The predominant linear NHEJ DNA products detected were dimers, followed by trimers and then multimers, for the Bgl II-digested substrates (upper panel of Fig. 1C). The amounts of these products formed were dose-dependent to the concentrations of nuclear proteins added to the assay. However, no NHEJ DNA products were formed until the SmaI-digested substrates were incubated with more than 15 µg 293T cell nuclear proteins (lower panel of Fig. 1C and 1D). In addition, only the dimeric NHEJ DNA products were produced for the SmaI-digested substrates. These findings suggest that DNAs with cohesive ends can be repaired more efficiently than those with blunt ends in the cell free *in vitro* NHEJ assay. In addition, as it would be expected that the formation of the NHEJ DNA products requires Ku70, K80 and DNA-PKcs [10], the nuclear proteins from *DNA-PKcs* null SCID mouse BM cells and these from immortalized *DNA-PKcs* ^−/−^, *Ku70* ^−/−^, and *Ku80* ^−/−^ mouse fibroblast cells failed to generate any of these NHEJ DNA products after they were incubated with the Bgl II-digested substrates (Fig. 2A and B). Complementation of *DNA-PKcs* ^−/−^ cell nuclear proteins with DNA-PKcs purified from HeLa cells produced NHEJ ligated DNA (Fig. 2C). Therefore, these results confirm that the ligated DNA products were generated through NHEJ.

### Detection and quantification of NHEJ DNA products by qPCR

**Figure 3:**
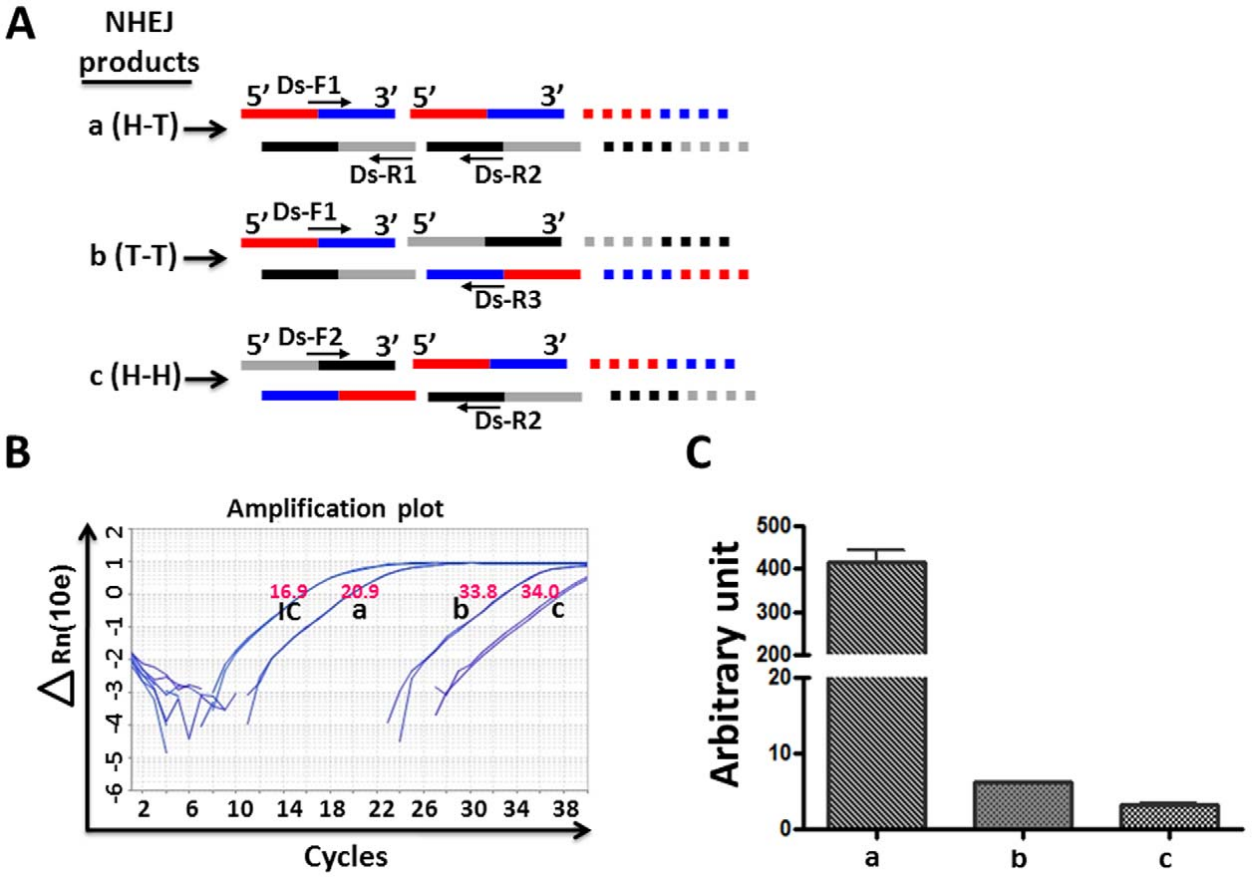
qPCR analysis of NHEJ activity. **A.** A diagram to illustrate the strategy for how to amplify various NHEJ products by qPCR with their respective pairs of primers. H-T (product *a*), T-T (product *b*), and H-H (product *c*) represent different NHEJ products with the orientations of head-to-tail, tail-to-tail, and head-to-head ligations of the Bgl II-digested linearized pDsRed2ER plasmid substrates, respectively. **B.** An example of qPCR analysis of different NHEJ products after the Bgl II-digested linearized pDsRed2ER plasmid substrates were incubated with 8 µg of 293T cell nuclear protein extracts. Products *a* (H-T), *b* (T-T) and *c* (H-H) were amplified with the primers of Ds-F1 and Ds-R2, Ds-F1 and Ds-R3, and Ds-F2 and DS-R2, respectively. The internal control (IC) for all ligated and non-ligated pDsRed2ER plasmid DNAs was amplified by the primers of Ds-F1 and Ds-R1. **C.** The arbitrary units for all NHEJ products analyzed by qPCR are presented as mean ± SD (n = 3 independent assays). The arbitrary units were calculated by the comparative C_T_ method (arbitrary units = 2^[−ΔCT]^ while ΔCT = C_T-Product_−C_T-IC_). a, *p*<0.05 vs. product *b*; b, *p*<0.05 vs. product *c*.

**Figure 4:**
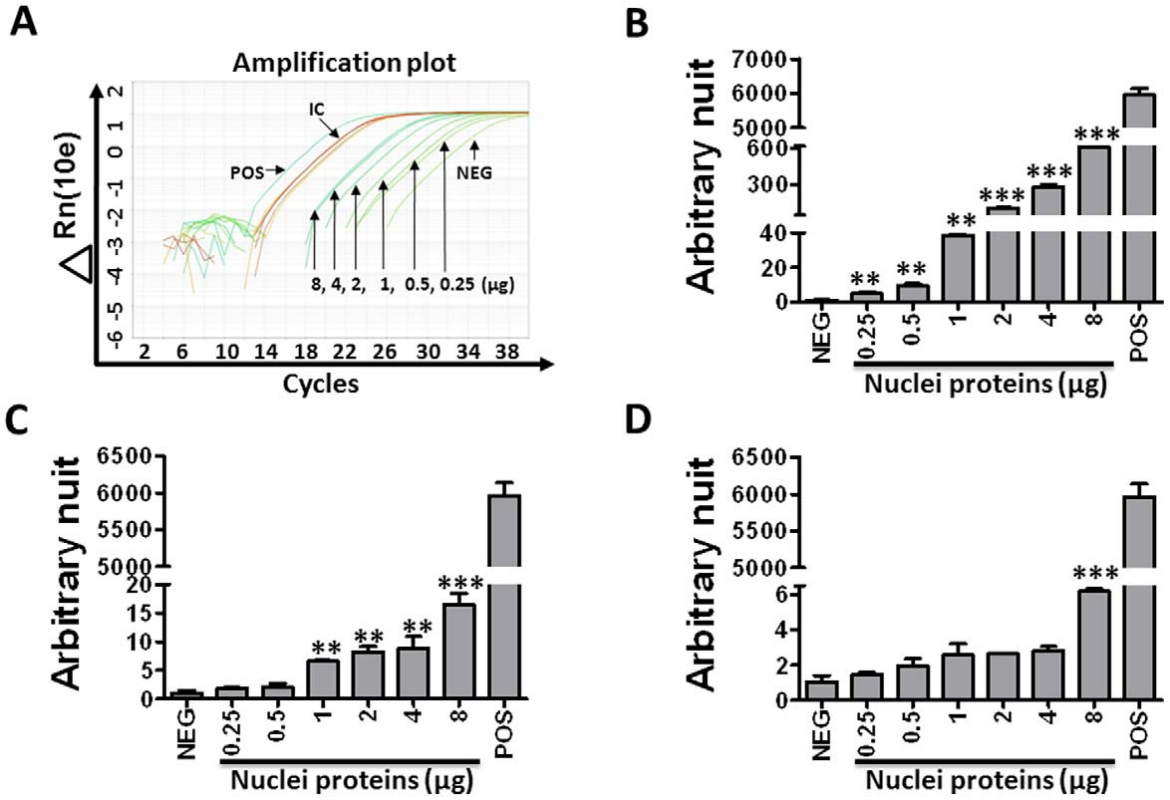
Dose-dependent production of NHEJ products. **A.** An example of qPCR analysis of product *a* after the Bgl II-digested linearized pDsRed2ER plasmid substrates were incubated with increasing concentrations of 293T cell nuclear protein extracts. A negative control (digested plasmid DNA without nuclear protein, NEG) and a positive control (undigested plasmid DNA, POS) were also included in the assay. **B–D.** The arbitrary units for NHEJ product *a* (**B**), *b* (**C**), *c* (**D**) were calculated as described in Figure 3 and presented as mean ± SD (n = 3). **p<0.01, ***p<0.001 vs. 0.25 µg proteins.

**Figure 5:**
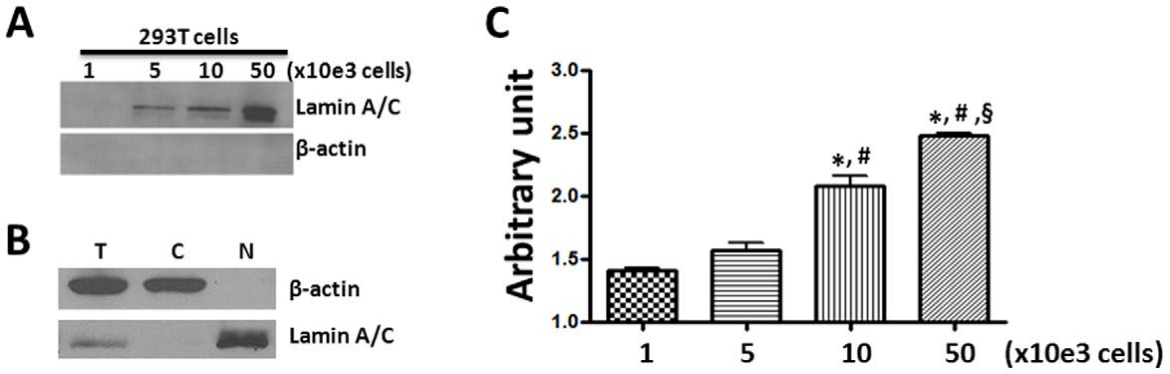
Sensitive detection of NHEJ activity in 293T cell nuclear protein extracts by the qPCR cell free *in vitro* NHEJ assay. **A.** Nuclear protein extracts from 1,000–50,000 293T cells were immunoblotted with an antibody against Lamin A/C. β-actin blot is included as a control to detect cytoplasmic protein contamination. **B.** The purity of the nuclear protein extracts (N) was further confirmed by Western blot with antibodies against Lamin A/C and β-actin in comparison with total cell lysates (T) and cytoplasmic protein extracts (C). **C.** qPCR analysis of product *a* after the Bgl II-digested linearized pDsRed2ER plasmid substrates were incubated with the nuclear proteins extracted from increasing number of 293T cells. The data are presented as mean ± SD of arbitrary units (n = 3 independent assays). The symbols of *, # and § represent *p*<0.05 vs. nuclear proteins from 1,000, 5,000, and 10,000 cells, respectively.

As shown in Fig. 3A, linearized pDsRed2ER plasmid substrates can be ligated by NHEJ in three different orientations, e.g. head-to-tail (H-T or product *a*), tail-to-tail (T-T or product *b*), and head-to-head (H-H or product *c*). Each of them can be specifically detected by the pairs of primers Ds-F1 and Ds-R2, Ds-F1 and Ds-R3, and Ds-F2 and Ds-R2, respectively. In addition, all ligated and non-ligated pDsRed2ER plasmid DNAs can be amplified by the Ds-F1 and Ds-R1 primers, which was included in every qPCR reaction as an internal control for normalization. Data presented in Fig. 3B are an example of the qPCR reactions. An arbitrary unit was calculated for each NHEJ DNA product after normalization with the internal control (Fig. 3C). The data showed that product *a* was the most abundant NHEJ products in the cell free *in vitro* NHEJ assay, followed by product *b* and then product *c*. In addition, the production of all these products was dose-dependent to the concentrations of the nuclear proteins used in the assay (Fig. 4A–D). Based on these findings, we selected product *a* for the qPCR assay to analyze NHEJ activities of nuclear proteins in the subsequent experiments and found that this assay is highly sensitive because it could measure NHEJ activities in 1 µg 293T cell nuclear proteins (Fig. 4) and nuclear extracted from as low as a few thousands of 293T cells (Fig. 5).

### Human and mouse HSCs and HPCs exhibit different proficiencies in the repair of DSBs

**Figure 6:**
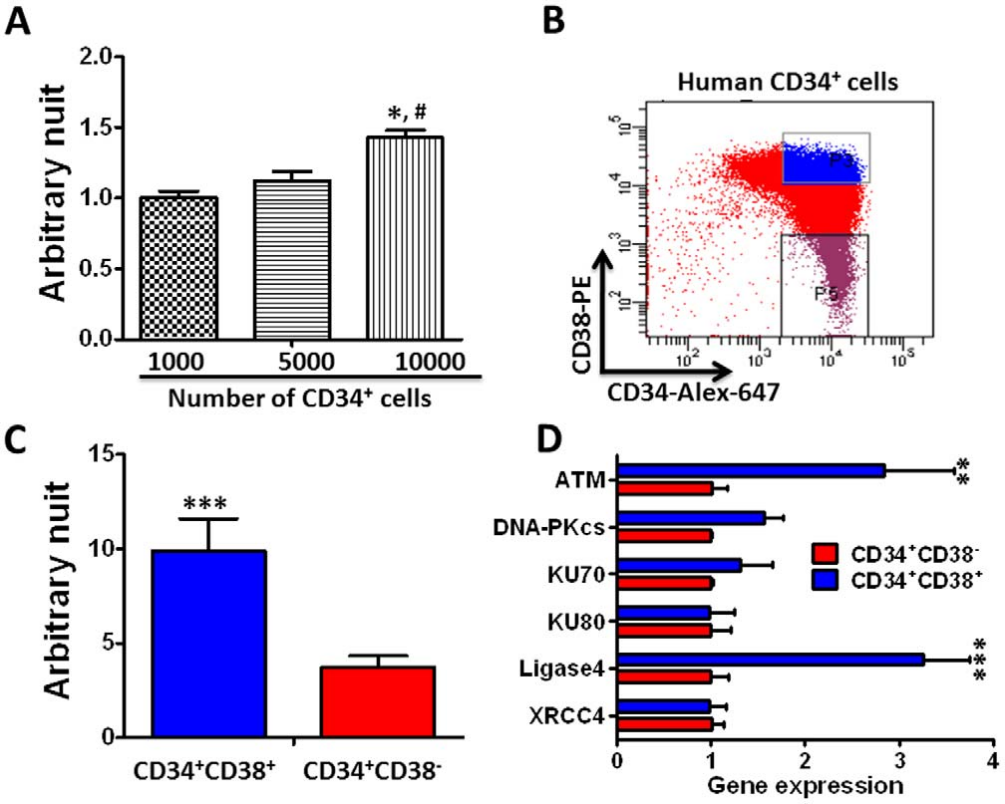
Nuclear protein extracts from human BM HPCs have higher NHEJ activity than those from HSCs. **A.** qPCR analysis of the NHEJ activity in nuclear proteins extracted from different numbers of human BM CD34^+^ cells. The data are presented as mean ± SD of arbitrary units (n = 3). The symbols of * and # represent *p*<0.05 vs. nuclear proteins from 1,000 and 5,000 cells, respectively. **B.** A representative gating strategy to sort human HSCs (CD34^+^CD38^−^ cells) and HPCs (CD34^+^CD38^+^ cells) from human BM CD34^+^ cells. **C.** NHEJ activity in nuclear proteins extracted from the same number (6,600) of human HSCs and HPCs. The data are presented as mean ± SD of arbitrary units (n = 3 independent experiments). **D.** Analysis of the expression of ATM, DNA-PKcs, KU70, KU80, Ligase 4, and XRCC4 mRNA in human BM HSCs and HPCs by qRT-PCR. The data are presented as ratios between HPCs and HSCs (n = 3 independent experiments). **p<0.01 and ***p<0.001 vs. HSCs.

Recently, Milyavsky et al. used the γH2AX foci and neutral comet assays to compare the ability of human cord blood HSCs and HPCs to repair DSBs generated by ionizing radiation (IR) and found that human HPCs repaired DSBs more rapidly than HSCs [12]. However, the mechanisms underlying the difference between human HSCs and HPCs are not known. Therefore, we first examined if we can confirm the finding using our newly developed qPCR-based cell free *in vitro* NHEJ activity assay. As shown in Fig. 6A, this assay is very sensitive as it could measure the NHEJ activities in nuclear proteins extracted from a few thousands of human BM CD34^+^ hematopoietic cells. Nuclear protein extracts from the same number of human BM HPCs (CD34^+^CD38^+^ cells) generated significantly more NHEJ DNA products than those from human BM HSCs (CD34^+^CD38^−^ cells) in the cell-free *in vitro* NHEJ assay (Fig. 6B & C), confirming that human HPCs indeed have a higher level of NHEJ activity than HSCs. This difference can be attributed at least in part to their differential expression of *LIG4*, as qRT-PCR analysis revealed that human HPCs expressed significantly higher levels of ligase 4 mRNA than HSCs (Fig. 6D)

**Figure 7:**
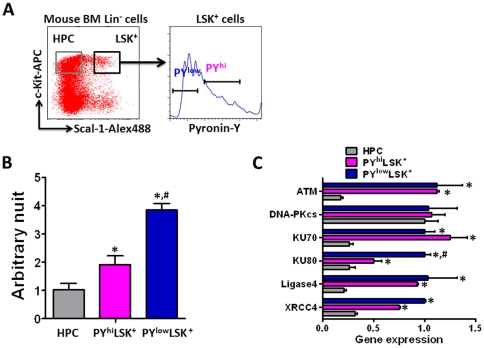
Nuclear protein extracts from mouse BM HSCs have higher NHEJ activity than those from HPCs. **A.** A representative gating strategy to sort HPCs (LKS^−^ cells) and cycling (PY^hi^LKS^+^ cells) and quiescent HSCs (PY^low^LKS^+^ cells) from mouse BM lineage negative cells (Lin^−^ cells). **B.** NHEJ activity in nuclear proteins extracted from the same number (20,000) of mouse HPCs and cycling and quiescent HSCs. The data are presented as mean ± SD of arbitrary units (n = 3). **C.** Analysis of the expression of ATM, DNA-PKcs, KU70, KU80, Ligase 4, and XRCC4 mRNA in mouse BM HPCs and cycling and quiescent HSCs by qRT-PCR. The data are presented as ratios between HPCs and HSCs (n = 3 independent assays). The symbols of * and # represent p<0.01 vs. HPCs and PY^hi^LKS^+^ cells, respectively.

In contrast, Mohrin et al. recently reported that mouse BM HSCs, particularly those in quiescence, repaired IR-induced DSBs more efficiently via the NHEJ pathway than mouse BM HPCs using the γH2AX foci and alkaline comet assays [13]. These findings raise an intrigue question whether there is a species difference between human and mouse HSCs and HPCs in their ability to repair DSBs [14], [15]. Therefore, we also compared the NHEJ activities of nuclear protein extracts from mouse BM HPCs (LKS^−^ cells), cycling HSCs (PY^hi^LSK^+^ cells) and quiescent HSCs (PY^low^LSK^+^ cells) using our qPCR cell free *in vitro* NHEJ assay (Fig. 7A & B). As shown in Fig. 7B, it was found that the nuclear protein extracts from both cycling and quiescent mouse BM HSCs had a significantly higher level of NHEJ activity than those from mouse BM HPCs. This finding is in agreement with the observation that both cycling and quiescent mouse BM HSCs expressed significantly great levels of KU70, KU80, ligase 4 and XRCC4 mRNA than HPCs (Fig. 7C). In addition, using IR-induced DSB foci assays Mohrin et al. showed that quiescent mouse BM HSCs exhibited a higher NHEJ activity than the HSCs that were in cell cycle and proliferating after they were activated by cytokines or mobilized by cyclophosphamide/granulocyte colony-stimulating factor treatment [13]. This finding is in agreement with our observation that the nuclear protein extracts from quiescent mouse BM HSCs had about 2-fold greater NHEJ activity than those from cycling mouse BM HSCs (Fig. 7C). These results demonstrate that our qPCR cell free *in vitro* NHEJ assay can be used to sensitively and accurately measure NHEJ activity in both human and mouse HSCs.

## Discussion

Currently, the γH2AX foci assay is the most widely used method to analyze DSBs and DSB repair [20]. It is several orders of magnitude more sensitive than the neutral comet assay and pulsed-field gel electrophoresis (PFGE) [20]. Therefore, the γH2AX foci assay has been widely used to analyze DSB repair in HSCs [12], [13]. However, the γH2AX foci assay is an indirect assay for DSBs, because the appearance and disappearance of γH2AX foci depend on the phosphorylation and dephosphorylation cascades that sense DSBs and mediate DSB repair and lag behind the occurrence of the actual damage and repair [20]. In addition, the formation and resolution of γH2AX foci can be affected by many other factors [20]. Therefore, validation of the results from the γH2AX foci assay by a method that can direct measure DSBs is always warranted. However, this cannot be accomplished by the use of the neutral comet assay and pulsed-field gel electrophoresis (PFGE) for HSCs, because they can only reliably detect tens or hundreds of DSBs which rarely occur in HSCs under a physiological condition or after exposure to stress without killing the cells instantly [20]. A cell free *in vitro* NHEJ assay has the potential to fill the gap by directly measuring the ability of HSCs to repair DSBs. This is because the majority of adult HSCs are quiescent and quiescent HSCs primarily repair DSBs through the NHEJ pathway due to a lacking of sister chromatids as the substrates for HR [13].

Unfortunately, the conventional cell free *in vitro* NHEJ assay is insensitive and requires tens µg nuclear proteins or nuclear extracts from a large number of cells for the assay. This is very difficult to achieve for HSCs and other tissue stem cells considering the scarcity of these cells in the body. As an example, Sotiropoulou et al. recently reported to use 40 µg nuclear proteins from CD34^+^ hair follicle bulge stem cells and CD34^−^ non-stem cells to perform the conventional cell free *in vitro* NHEJ assay in order to confirm that the stem cells have a higher DSB repair activity than CD34^−^ non-stem cells [21]. It is likely that to obtain such a large quantify of nuclear proteins requires isolation of hundreds of thousands or even millions of CD34^+^ hair follicle bulge stem cells from a large number of mice. Therefore, developing a sensitive and quantitative cell free *in vitro* assay that can be used to measure NHEJ activity in a few thousands of cells is highly desirable for studying DSB repair function of HSCs.

Here, we report the development of a qPCR-based cell free *in vitro* NHEJ assay using linearized plasmids as the substrates and qPCR technique. We showed that the linearized pDsRed2ER plasmids with cohesive ends generated by BglII were better substrates for the cell free *in vitro* NHEJ assay than those with blunt ends produced by SmaI. This assay is highly specific for NEHJ, because the formation of the ligated DNA products in the cell free *in vitro* NHEJ assay depends on DNA-PKcs, Ku70 and Ku80, as the nuclear proteins from *DNA-PKcs* ^−/−^, *Ku70* ^−/−^ and *Ku80* ^−/−^ cells failed to generate any of these NHEJ DNA products after they were incubated with the Bgl II-digested substrates. The NHEJ activity of the nuclear proteins extracted from *DNA-PKcs* ^−/−^ cells was restored after complementation of the nuclear extracts with DNA-PKcs proteins purified from HeLa cells. Interestingly, the most of the NHEJ DNA products generated in this assay were linear and had a head-to-tail (H-T) orientation. Therefore, we could acurately measure NHEJ activity in cell nuclear extracts by quantifying the abundance of the H-T products (product *a*) using qPCR. We showed that we could measure NHEJ activity in less than 1 µg 293T cell nuclear proteins with this qPCR-based cell free *in vitro* NHEJ assay. In addition, we could quantify NHEJ activity in nuclear extracts from a few thousands of human BM CD34^+^ hematopoietic cells using this assay. Therefore, this qPCR-based NHEJ assay is much more sensitive than the conventional cell free *in vitro* NHEJ assay.

More importantly, using this assay, we found that the nuclear protein extracts from human HSCs had a lower level of NHEJ activity than those from HPCs. In contrast, it was found that the nuclear extracts from both mouse cycling (PY^hi^LKS^+^ cells) and quiescent HSCs (PY^low^LKS^+^ cells) had a higher level of NHEJ activity than those from cycling HPCs (LKS^−^ cells). Because almost all HPCs and the cycling HSCs isolated from mouse BM by cell sorting are PY^hi^ cells and in the cell cycle [19], the difference between mouse BM HSCs and HPCs cannot be simply ascribed to a difference in their cell cycle status. These findings confirm that human and mouse HSCs and HPCs have different abilities to repair DSBs via NHEJ as shown in the recent studies [12], [13]. The difference in the abilities of human and mouse HSCs and HPCs to repair DSBs through NHEJ is likely attributed to their differential expression of key NHEJ DNA damage repair genes, such as *LIG4*. This is because it was found that human HSCs expressed less mRNA for ligase 4 than human HPCs, while mouse HSCs expressed higher levels of ligase 4 mRNA than mouse HPCs. In addition, both cycling and quiescent mouse HSCs also expressed higher levels of KU70, KU80 and XRCC4 mRNA than mouse HPCs. The mechanisms underlying the differential expression of these DSB repair genes in human and mouse HSCs and HPCs are unknown at the present. Similarly, the biological significance of these differences has yet to be determined.

In summary, the findings presented in this manuscript suggest that the qPCR-based cell free *in vitro* NHEJ assay we developed can be used to sensitively measure the ability of HSCs to repair DSBs. This assay can also be applied to study DSB repair in other populations of rare tissue stem cells. In addition, it could help us to gain more insights into the mechanisms by which HSCs and tissue stem cells maintain their genomic stability.
